# Efficacy, pharmacokinetics and safety of liposomal synthetic cannabidiol injected subcutaneously in dogs: a randomized, blinded, placebo-controlled, crossover clinical trial

**DOI:** 10.3389/fvets.2025.1746266

**Published:** 2026-01-19

**Authors:** Yael Shilo-Benjamini, Joshua Milgram, Eran Lavy, Maxim Quint, Dinorah Barasch, Ahuva Cern, Yechezkel Barenholz

**Affiliations:** 1 Laboratory of Membrane and Liposome Research, Department of Biochemistry, Hadassah Medical School, The Hebrew University of Jerusalem, Jerusalem, Israel; 2 Koret School of Veterinary Medicine, The Robert H. Smith Faculty of Agriculture, Food and Environment, The Hebrew University of Jerusalem, Rehovot, Israel; 3 The Mass Spectrometry Unit, School of Pharmacy, The Hebrew University of Jerusalem, Jerusalem, Israel

**Keywords:** analgesia, canine, CBD, liposomes, osteoarthritis, pharmacokinetics, prolonged release, synthetic cannabidiol

## Abstract

**Background:**

Cannabidiol (CBD) has anti-nociceptive and anti-inflammatory characteristics, and was reported to provide analgesia in dogs with osteoarthritis. However, oral CBD has poor bioavailability due to significant first-pass hepatic metabolism. Encapsulation of CBD into liposomes was reported by our group to facilitate CBD slow-release, and provide high bioavailability and analgesia in a pilot study in dogs with osteoarthritis.

**Objectives:**

To determine the pharmacokinetics and effects of liposomal-synthetic-cannabidiol (L-sCBD) injection compared with placebo in dogs with radiographically confirmed naturally-occurring osteoarthritis.

**Animals:**

Eight client-owned dogs (4 males, 4 females; 8.5 [4.5–12.5] years-old; 34.9 [22.7–42.7] kg).

**Methods:**

Dogs were injected subcutaneously twice with a 4-week interval; once with 7 mg/kg L-sCBD (50 mg/mL) and once with empty liposomes of identical lipid composition (placebo; equivalent volume) in a randomized, blinded, crossover design. Each dog routine analgesics (e.g., non-steroidal anti-inflammatories) were continued. Blood was sampled for CBD and metabolites concentrations, complete blood count and serum chemistry up to 4-weeks post-injections. Efficacy was assessed *via* activity monitoring collar and scorings by owners and two veterinary specialists. Vital signs and local response were monitored. Data analysis used aligned rank transform ANOVA, permutation test and Wilcoxon signed-rank test (*p*-value < 0.05).

**Results:**

CBD plasma concentrations were detected up to 4-weeks; median peak plasma concentration (C_max_) was 58.2 [range 35.1–141.0] ng/mL, median time to C_max_ was 3 [3–7] days and median half-life 6.1 [4.6–9.5] days. The metabolites 6/7-hydroxy-CBD, and *7-*carboxy-CBD were detected at low concentrations. Pain and lameness scores and behavior were significantly improved after L-sCBD treatment *versus* placebo. At 3-days after L-sCBD treatment, neutrophils and alkaline-phosphatase increased significantly, while hematocrit and albumin decreased (all within reference range, except neutrophils in 2/8 dogs). Adverse effects included 2-days fever and a minor-moderate local swelling, which resolved spontaneously.

**Conclusions and clinical relevance:**

Subcutaneous L-sCBD provided long-term CBD plasma concentrations, improved analgesia and was tolerated by all dogs. A larger clinical cohort is required to further assess L-sCBD benefits and safety.

## 1 Introduction

Osteoarthritis is one of the most common musculoskeletal diseases in dogs, which affects life quality of both dogs and owners (1, 2). In recent years, cannabidiol (CBD), the non-psychoactive component of *Cannabis sativa*, has gained veterinary interest for its various therapeutic indications, including pain management (3–5). CBD has anti-nociceptive and anti-inflammatory characteristics and has been reported to provide analgesia for osteoarthritis in dogs (6–10). However, CBD is highly lipophilic, and its water solubility is poor (0.0627 μg/mL in milliQ water) (11, 12). Therefore, CBD is poorly absorbed from the gastrointestinal tract and undergoes significant first-pass liver metabolism, leading to a very low oral bioavailability (12–14).

Recently, our group reported an alternative, injectable route for CBD in dogs, using a liposomal delivery system (15, 16). Liposomes are vesicles made of one or more bilayers of well-characterized phospholipids. They are attractive for pharmaceutical application because this delivery system is biocompatible, biodegradable, and non-toxic (17–19). Additionally, the US Food and Drug Administration (FDA) has approved many liposomal drug-products (19). Prolonged-release injectable liposomal CBD formulation results in increased CBD bioavailability (12, 15, 20) and a more convenient administration route allowing better owner compliance (15, 16).

The objectives of this study were to investigate (1) the analgesic efficacy, (2) the pharmacokinetics, and (3) adverse effects of subcutaneous injectable liposomal synthetic CBD (L-sCBD) compared with placebo-control, using a crossover design in dogs with naturally-occurring osteoarthritis. Our hypotheses were that (1) L-sCBD will provide long-term analgesia of 3–4 weeks, (2) CBD will be detected in dogs’ plasma for at least 4-weeks, and (3) no major adverse effects will occur, although, minor local response at the injection site may be observed.

## 2 Methods

### 2.1 Study design and animal selection

This study was a prospective, single-site, randomized, blinded, placebo-controlled, crossover clinical trial, conducted at the Veterinary Teaching Hospital of Koret School of Veterinary Medicine, the Hebrew University of Jerusalem. Each dog served as its own control, and was injected once with the L-sCBD and once with placebo (empty liposomes), according to their allocated sequence. All dogs were client-owned, and during the study period (total of 8-weeks) and after study completion, dogs remained at their original homes and under the direct owners’ care.

The study was approved by the Hebrew University of Jerusalem Animal Care and Use Committee (IACUC; approval protocol MD-22-16886-2). Additionally, a signed informed-consent was obtained from all dog owners. The consent form was approved by the IACUC, and included study goals, explanation on CBD, possible adverse effects, detailed study protocol and required Hospital visits, and emergency contact number.

Inclusion criteria were dogs aged 2–13 years-old, with a body weight over 10 kg, and with clinically (orthopedic examination) and radiographically confirmed osteoarthritis in at least one joint. Exclusion criteria included dogs younger than 2 years or older than 13 years of age, with a body weight of less than 10 kg. Additionally, dogs were excluded if they were diagnosed with any liver disease, with severe heart or kidney diseases, or with any other severe systemic disease. Dogs were also excluded if they underwent a surgery in the 3-months prior to study initiation, were scheduled to undergo a surgery during the study period, or were diagnosed with an operatable orthopedic disease, and therefore, were referred to undergo a surgical procedure.

**Figure 1:**
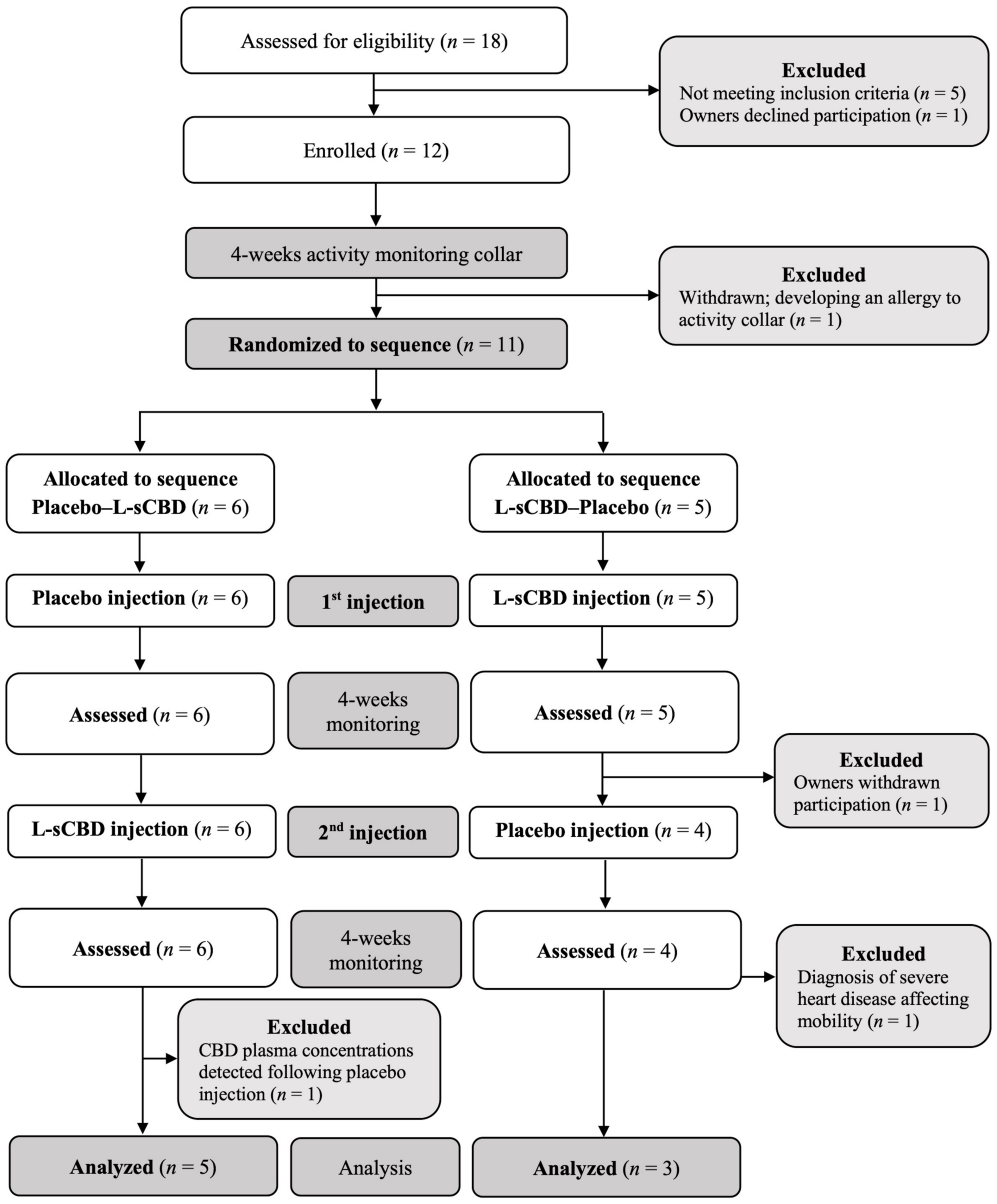
Consolidated standards of reporting trials (CONSORT) flow diagram for dogs with naturally occurring osteoarthritis, administered a subcutaneous injection of liposomal-synthetic-CBD (L-sCD; 7 mg/kg) or placebo (empty liposomes) at an equal volume in a randomized, blinded, crossover design.

Following enrollment, dogs were fitted with an activity monitoring collar (PetPace, Burlington, MA, United States) for at least 4-weeks prior to the first intervention. Sequence randomization of dogs was performed using excel RANDOM function with blocks of six (Microsoft Corporation, Redmond, Washington, United States), and dogs were allocated to sequence according to their recruitment order (Figure 1). On the morning of the first intervention, vital signs were recorded, dogs underwent physical examination, orthopedic examination by a specialist in small animal surgery, and blood was collected from a peripheral artery (cephalic or saphenous) for baseline values.

For ethical reasons, all dogs continued receiving their prescribed routine joint supplements and/or analgesics as before enrollment to the study. In order to increase dog welfare and to facilitate handling, procedures such as blood sampling and injections of L-sCBD/placebo were performed while the dogs were administered oral treats.

### 2.2 L-sCBD and placebo interventions

The L-sCBD and the placebo (empty liposomes) were obtained from Innocan Pharma™ (Herzliya, Israel). According to the product certificate of analysis, both formulations were prepared under strict aseptic conditions. Samples from L-sCBD and the placebo liposomes were submitted to Hy-Labs (Rehovot, Israel), a certified and accredited laboratory by the FDA and the Israeli Ministry of Health, to confirm that the formulations are sterile and below the approved limit of endotoxins. The results of these tests met the requirements of extra-vascular administered drugs in human.

The liposomes of both formulations were prepared of identical lipid composition from hydrogenated soy phosphatidylcholine (HSPC) (Lipoid GmbH, Ludwigshafen, Germany). The L-sCBD liposomes were loaded with synthetic CBD (absence of tetrahydrocannabinol [THC], purity > 98%; Purisys LLC., Athens, GA, United States) at a concentration of 50 mg/mL, while the placebo liposomes were used without loading. Both liposomal formulations had the same appearance.

Each dog was administered one L-sCBD injection at a dose of 7 mg/kg (0.14 mL/kg) and one placebo injection at an equal volume with a 4-week interval, according to the allocated sequence (Figure 1). Prior to injections, dogs were clipped at the shoulders area, and the skin was prepared aseptically with chlorhexidine and ethyl alcohol 70%. Injections were administered subcutaneously at the prepared skin area using a 21-gauge, 1-inch needle, with the second injection administered approximately 5 cm caudal to the clipped location of the first injection, which was still visible. Dogs were monitored closely for 4-weeks after each injection (Figure 1).

### 2.3 Monitoring

#### 2.3.1 Pharmacokinetics

##### 2.3.1.1 Quantification of CBD and its metabolites in plasma

One mL blood was collected for quantification of CBD and its metabolites in plasma at baseline, 6-h, 3-days, and weekly at 1–4 weeks following each of the two injections. Sampling frequency was determined based on a former pilot study (16), while accommodating owners’ willingness to cope with multiple hospital visits throughout the 8-weeks study period. Blood was collected into 1-mL ethylenediamine tetra-acetic acid (EDTA) tubes and centrifuged at room temperature, x 6,200 rpm, for 10 min (ScanFuge Mini, ScanSpeed, LaboGene, Lillerød, Denmark) to separate the plasma within 5 min from collection. Following separation, plasma was kept for 2–10 h at −20 °C and then moved to −80 °C until analysis.

CBD and its metabolites were quantified using UHPLC-tandem mass spectrometry (LC–MS/MS). The concentration-calibration curve was performed using three naïve dogs’ plasma (4-10-years-old), collected for this purpose. Plasma samples for CBD quantification were prepared by CBD extraction from the plasma and then spiking the samples with cannabigerol (CBG, 1 mg/mL in methanol; Sigma, Cat. C-141-1) as the internal standard (IS), followed by dilution of the plasma 5-fold in acetonitrile. After vigorous vortex it was centrifuged, and the upper phase was analyzed. Final IS concentration in the samples was 100 ng/mL. CBD concentrations were calculated based on a calibration curve of CBD in plasma having 100 ng/mL of IS. In case of low CBD concentrations, the upper phase after dilution in acetonitrile was evaporated to dryness and reconstituted with 100 μL of acetonitrile. In this case, CBD concentrations were calculated from a calibration curve prepared in the same way (21).

The materials used for LC–MS/MS included: LC/MS-grade acetonitrile, methanol and water (Biolab Ltd., Jerusalem, Israel), and LC/MS-grade formic acid (Fisher Chemical™, Optima™, Waltham, MA, United States). UHPLC–MS/MS analyses were conducted on a Sciex (Framingham, MA, United States) Triple Quad™ 5,500 mass spectrometer coupled with a Shimadzu UHPLC System (Kyoto, Japan). The chromatographic separations were performed on a CORTECS® (Waters Corp., Milford, MA, United States) column (C18, 2.7 μm particle size, 100 × 2.1 mm), protected by a VanGuard® (Waters Corp., Milford, MA, United States) pre-column. The injection volume was 10 μL, the oven temperature was maintained at 40 °C and the autosampler tray temperature was maintained at 5 °C. Gradient elution mobile phases consisted of phase A (0.1% formic acid in water) and phase B (acetonitrile). Gradient elution (300 μL/min) was held at 15% B for the first 0.5 min, followed by a linear increase toward 60% B in 6.5 min, a linear increase toward 75% B in 3 min, a linear increase toward 98% B in 2 min and held at 98% B for 3 min, before re-equilibrating for 3 min at initial conditions (21).

CBD and its metabolites were detected and quantified in positive and negative ion mode using electron spray ionization (ESI) and multiple reaction monitoring (MRM) mode of acquisition. CBG was used as the IS. Their transitions are shown in the Appendix. The TurboIonspray® probe temperature was set at 500 °C with the ion spray voltage at 5,500 V (for positive mode) and −4,500 V (for negative mode). The curtain gas was set at 25.0 psi. The nebulizer gas (Gas 1) was set to 50 psi, the turbo heater gas (Gas 2) was set to 60 psi and the collision gas (CAD) was set to 8 psi. The entrance potential (EP) was set at 10 V and -10 V, respectively. The collision energy potentials (CE), collision cell exit potentials (CXP) and declustering potentials (DP) for the monitored transitions are given in the Appendix. The dwell time was 15 milliseconds. Data acquisition was performed using Analyst 1.6.3 software and data was analyzed using MutiQuant 2.1 software, both distributed by Sciex. The limit of quantification (LOQ) was 0.5 ng/mL for CBD and its metabolites.

##### 2.3.1.2 Pharmacokinetic analysis

Pharmacokinetic analysis was performed for the L-sCBD injection using a non-compartmental analysis with Phoenix WinNonlin (Version 8.5.2, Certara™, NJ, United States). The last plasma sample used for all dogs was at 4-weeks post-injection. Calculated parameters included the peak plasma concentrations (C_max_), the time to C_max_ (T_max_). The area under the concentration–time curve (AUC) was calculated by the logarithmic trapezoidal method from time of dosing to the last time point of blood sampling. The area under the moment curve (AUMC) was calculated from the time of dosing to the last measurable point. Mean residence time (MRT) was calculated by AUMC/AUC and referred to the time of dosing until the time of the last measurable concentration. The terminal slope (*λ*) was estimated by linear regression through the last time points (minimum of 3) and used to calculate the terminal half-life from the following equation: half-life = 0.693/λ.

#### 2.3.2 Blood work

Blood samples were collected at baseline and then at 3-days, 1- and 4-weeks after each of the two injections. Approximately 1-mL of the blood was placed in EDTA tubes for complete blood count (CBC) (ADVIA 2120i Hematology System, Siemens Healthineers, Erlangen, Germany); white blood cells (WBC), neutrophils, lymphocytes, monocytes, eosinophils, basophils, red blood cells (RBC), hematocrit, hemoglobin, mean corpuscular volume (MCV), mean corpuscular hemoglobin (MCH), mean corpuscular hemoglobin concentration (MCHC), reticulocytes, platelets, plateletcrit (PCT), mean platelet volume (MPV), platelets distribution width (PDW).

Two-mL of blood were placed in tubes containing a separator gel (CAT Serum Sep Clot Activator, Vacuette®, Greiner Bio-One, Kremsmünster, Austria) for serum chemistry (cobas® 6,000, Roche Diagnostics Corporation, Indianapolis, IN, United States); creatine kinase (CK), aspartate aminotransferase (AST), alanine aminotransferase (ALT), alkaline phosphatase (ALP), gamma-glutamyl transpeptidase (GGT), amylase, triglycerides, cholesterol, total bilirubin, glucose, albumin, total protein, urea, creatinine, phosphate, total calcium, sodium, chloride, potassium and CO_2_.

#### 2.3.3 Body weight and vital signs

Body weight was recorded at baseline before the first injection, and then weekly (a total of 8-weeks) (Digital veterinary weighing scale, Foschi Srl, Rome, Italy). Vital signs were measured at baseline, 6-h, 3-days, and then weekly at 1–4 weeks following each injection. Vital signs included heart rate (HR) using a stethoscope placed over the heart, respiratory frequency (*f*_R_) by observing thoracic movements, rectal temperature (RT) using digital thermometer and non-invasive mean arterial blood pressure (MAP; CASMED 740; CAS Medical Systems Inc., Branford, CT, United States). MAP was measured by placing the cuff on the antebrachium/forearm when the dog was in sternal recumbency.

#### 2.3.4 Behavior, activity monitoring collar, lameness and pain assessment

Changes in dog behavior were reported weekly by the owners. The PetPace activity monitoring collar collected data from the dogs for at least 4-weeks prior to the first injection and then 4-weeks after each of the two injections. At study completion, data recorded over 12-weeks from the PetPace cloud platform was downloaded and divided into three periods: (1) 4-weeks before the first injection (baseline), (2) 4-weeks after the L-sCBD injection and (3) 4-weeks after the placebo injection. For each dog the mean weekly score of 6 parameters was calculated; activity, position and sleep scores, calories spent, pulse rate and heart rate variability [HRV].

Lameness was scored using a visual lameness scale (VLS) previously reported in dogs with osteoarthritis (22, 23). The scale included 6 scores; 0–5, where 0 = sound; normal limb use, 1 = detectable lameness, 2 = mild weight bearing lameness, 3 = moderate weight bearing lameness, 4 = severe weight bearing lameness, and 5 = non weight bearing lameness. One specialist in small animal veterinary surgery (JM), blinded to the treatment sequence, performed orthopedic exams and scored VLS of all dogs in the study at baseline and then every-other-week. A pain interactive visual analog scale (iVAS) was used for veterinary assessment with a scale of 0–10; 0 = no pain, 10 = worse possible pain. Two blinded to treatment veterinary specialists scored all dogs at baseline and then every week (veterinary anesthesia and analgesia specialist; YSB) or every-other-week (small animal veterinary surgery specialist; JM). Due to a lack of a validated scale in the owners’ native language, a simple descriptive scale of 1–5 was used for owner assessment of dog quality of life (QoL); 1 = poor, 2 = fair, 3 = good, 4 = very good and 5 = excellent. This simple scale is a part of the Canine Brief Pain Inventory (CBPI) (24). Owners were similarly blinded and scored their dogs at baseline before the first injection and then once weekly for 4-weeks after each injection (total of 8-weeks).

#### 2.3.5 Adverse effects and follow-up

A veterinarian (YSB) recorded vital signs and monitored each dog closely for adverse local and systemic reactions for the first 6-h, at 3-days, and then once-weekly during the 4-week monitoring period after each injection. Additionally, dogs were monitored at home by their owners for the duration of the study.

Following study termination, dog owners were contacted by phone every other month for 6-months, and subsequently every 4–6 months until manuscript submission. Additionally, owners were requested to inform the attending veterinarian of any health status changes.

### 2.4 Statistical analysis

Data analysis was performed using R Programming (version R 4.2.0, R studio, R Foundation for Statistical Computing, Vienna, Austria). Analysis methods included non-parametric testing using Aligned Rank Transform ANOVA [for the period of 4-weeks together] and permutation tests. When significant change was detected, then the Wilcoxon signed-rank test was used to compare the treatments (L-sCBD *versus* placebo) at each time point. Formal normality testing was not performed in this study, because all inferential analyses were conducted using non-parametric methods, which do not assume normally distributed data. This choice was made *a priori* due to the small sample size. Additionally, the limited sample size precluded reliable stratified or covariate-adjusted analyses of sex and age. Because sample size was small, descriptive statistics are expressed as median (range as minimum-maximum). A *p*-value < 0.05 was considered significant.

## 3 Results

### 3.1 Animals

Eighteen dogs were evaluated for eligibility, and six of them were immediately excluded for the following reasons. In one case, owners declined participation, two dogs were diagnosed with an operatable orthopedic disease and were referred for an elective surgical treatment (one dog with cranial cruciate ligament rupture and the other with bilateral patellar luxation), three dogs were diagnosed with severe systemic diseases that were found to be the cause of “deterioration” in their osteoarthritic condition (one dog with Addison’s disease, one with suspected carcinoma and lung metastases, and one with a neuromuscular disorder). Following enrollment of 12 dogs, one dog became allergic to the PetPace collar and was withdrawn from the study prior to sequence allocation. The 11 remaining dogs were allocated to one of the two study sequences (Figure 1). Of these 11 dogs, 3 were withdrawn or excluded: one owner terminated participation due to a local adverse response at injection site. The second dog had a known mild heart disease, and presented with several non-orthopedic related mobility deterioration episodes, including one before the first injection. These episodes were correlated with tachycardia alerts by the PetPace monitoring collar, and were later diagnosed as ventricular tachycardia. The third dog had CBD of an unknown source detected in its plasma samples following the first placebo injection (Figure 1). Demographic data, joints affected and routine treatments of the 8 dogs that completed the study are presented in Table 1.

**Table 1:** Demographic data, joints affected and routine treatments of 8 dogs suffering from osteoarthritis, allocated to a subcutaneous liposomal-synthetic-cannabidiol (L-sCBD) and placebo liposomes injections with 4-weeks interval in a randomized manner.

Dog	Sex	Age (years)	Breed	Body weight (kg)	Affected joints	Supplements and analgesics	Other health conditions
1	Neutered male	8	Mixed	32	Hip, stifles, tarsuses, elbows, carpuses	Omega 3, firocoxib (every other day), dipyrone (as needed)	Gastrointestinal sensitivity, severe muscle atrophy
2	Neutered male	7.5	Mixed	23	Hip	Omega 3, glucosamine, chondroitin, carprofen, dipyrone (as needed)	Lumbar pain
3	Neutered male	5	Mixed	41.7	Stifles, tarsuses, elbows	Omega 3, glucosamine, chondroitin, firocoxib	Bilateral femur head excision at 1-year-old
4	Neutered male	12.5	Mixed	42	Hip, stifles, carpuses	Omega 3, glucosamine, Coenzyme Q10, Amorphous calcium carbonate	Kidney disease, obese
5	Spayed female	9	Malinois	31.7	Hip, stifles, tarsuses, carpuses	Firocoxib, gabapentin	Left pelvic limb mass (suspected sarcoma; distal femur level)
6	Spayed female	4.5	Mixed	42.6	Hip, stifles, right elbow	Glucosamine	Bilateral carpal over extension, obese
7	Spayed female	11	Mixed	35	Hip, right shoulder	Omega 3, probiotics, WeConfort	Kidney disease, mildly increased liver enzymes
8	Spayed female	10.5	Golden retriever	32	Hip, stifles	Omega 3, probiotics, gabapentin, WeConfort, bedinvetmab (Librela) injections	Hypothyroidism (treated with Levothyroxine), laryngeal paralysis

### 3.2 Pharmacokinetic data

**Figure 2:**
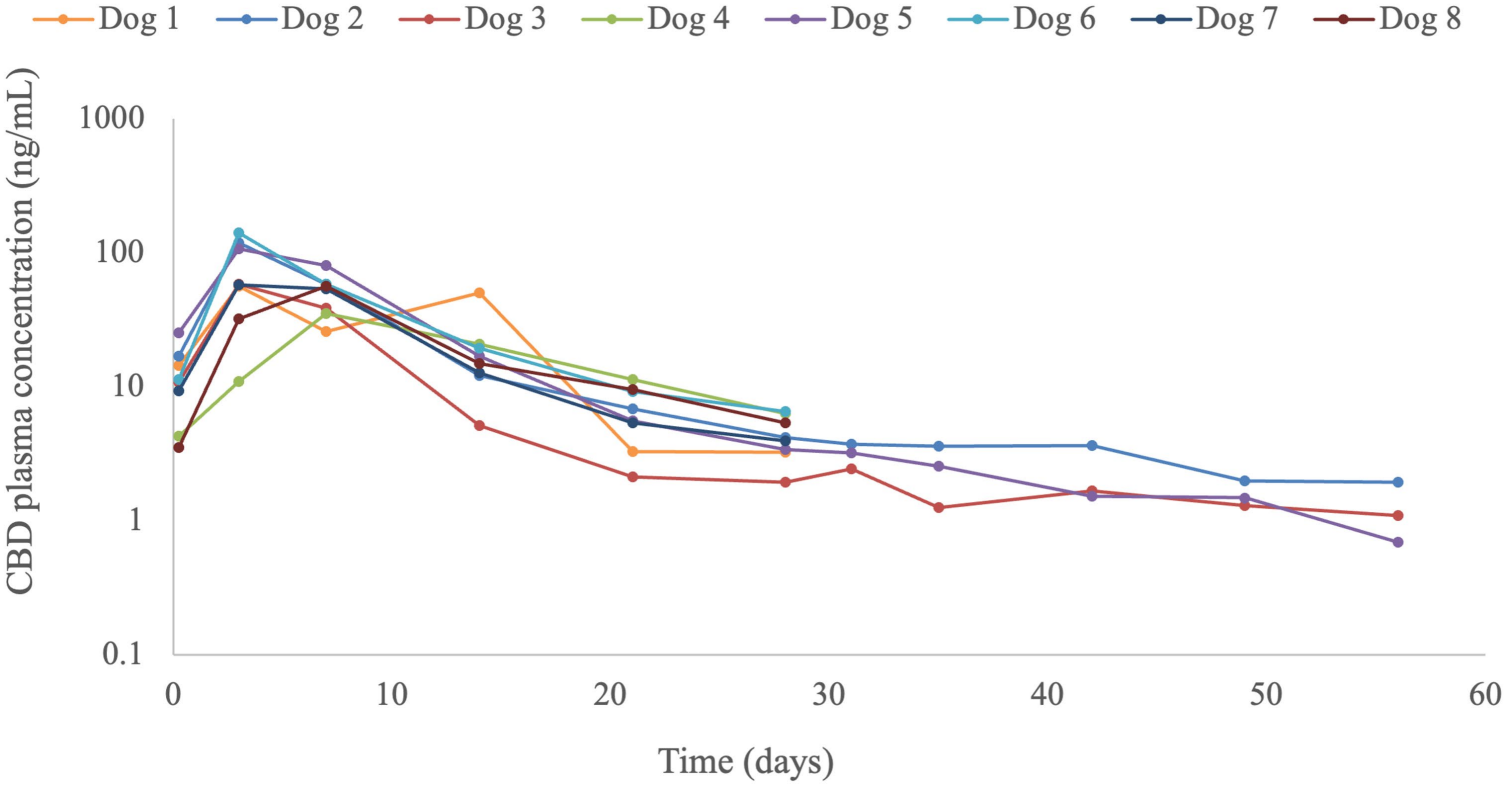
Cannabidiol (CBD) plasma concentrations (ng/mL) from eight dogs after a liposomal-synthetic-CBD (L-sCD; 7 mg/kg) subcutaneous injection. Plasma was collected up to 4 weeks post-injection (*n* = 5) or up to 8 weeks post-injection (*n* = 3).

CBD plasma concentrations were detected in all dogs throughout the monitoring period; with a concentration of 4.1 (1.9–6.5) ng/mL at 4-weeks (*n* = 8 dogs), and a concentration of 1.5 (0.7–2.5) ng/mL at 8-weeks (*n* = 3 dogs) post-L-sCBD-injection (Figure 2). Pharmacokinetic analysis was performed using 4-weeks post-L-sCBD-injection data from all dogs (Table 2). CBD plasma profile showed an increase up to C_max_ at 3-days (6 dogs) or 7-days (2 dogs), and then decreased slowly. The half-life calculated for 4 weeks data had a median of 6.1 days (range 4.6–9.5). However, for dogs no. 2, 3 and 5 that received the L-sCBD injection first, CBD quantification was available for 8-weeks. The half-life for these 3 dogs was calculated using 6-weeks data and resulted in longer half-lives of 15.6, 8.4 and 7.3 days, respectively (for dog no. 3 lambda <0.8).

**Table 2:** Pharmacokinetic data of plasma cannabidiol (CBD) from eight dogs after liposomal-synthetic-CBD (L-sCBD; 7 mg/kg) subcutaneous injection.

Dog	C_max_ (ng/mL)	T_max_ (days)	Half-life (days)	AUC (ng·d/mL)	AUC/dose (ng·d/mL/mg/kg)	MRT (days)
1	56.1	3	5.4^#^	740	105.7	9.9
2	118.2	3	9.1	902	128.9	6.6
3	58.6	3	4.9	492	70.3	6.2
4	35.1	7	8.2	491	70.1	11.9
5	106.7	3	4.6	1,011	144.4	6.9
6	141.0	3	6.6	1,039	148.4	7.3
7	57.8	3	5.6	645	92.1	7.9
8	56.6	7	9.5	615	87.9	9.6
Median	58.2	3	6.1	693	98.9	7.6

The calculations are based on blood samples collected for 4-weeks post-injection.

^#^Lambda < 0.8.

C_max_, peak plasma concentration; T_max_, time to maximum plasma concentration; AUC, area under the concentration–time curve; MRT, mean residence time.

Four CBD metabolites; 6α-hydroxy-CBD (6α-OH-CBD), 6β-hydroxy-CBD (6β-OH-CBD), 7-hydroxy-CBD (7-OH-CBD) and *7-*carboxy-CBD (7-COOH-CBD) were detected at low concentrations and for a shorter duration than the parent drug. The males showed only 1–3 of the metabolites up to 7-days post-L-sCBD-injection, while all four metabolites were detected in all females, and 6α-OH-CBD was detected up to 14-days (Table 3).

**Table 3:** Plasma concentrations of cannabidiol (CBD) metabolites: 6α/6β hydroxy-CBD (6α/6β-OH-CBD), 7-hydroxy-CBD (7-OH-CBD) and *7-*carboxy-CBD (7-COOH-CBD) detected in dogs after liposomal-synthetic-CBD (L-sCBD; 7 mg/kg) subcutaneous injection.

Time	6α-OH-CBD (ng/mL)	6β-OH-CBD (ng/mL)	7-OH-CBD (ng/mL)	7-COOH-CBD (ng/mL)
Baseline	< LOQ	< LOQ	< LOQ	< LOQ
6-h	2.7 (*f* = 1)	< LOQ	< LOQ	0.7 (*f* = 1)
3-days	6.7 (1.4–20.5) (*f* = 4; *m* = 4)	1.5 (0.6–5.0) (*f* = 4; *m* = 1)	1.8 (0.7–4.0) (*f* = 4; *m* = 2)	9.3 (6.0–14.4) (*f* = 4; *m* = 2)
1-week	8.8 (2.8–31.0) (*f* = 4; *m* = 3)	1.1 (0.9–4.4) (*f* = 4)	1.0 (0.6–2.6) (*f* = 3)	3.2 (1.1–5.8) (*f* = 4)
2-weeks	1.9 (1.2–6.4) (*f* = 4)	< LOQ	< LOQ	1.2 (0.9–1.4) (*f* = 2)
3-weeks	1.4 (0.8–1.9) (*f* = 2)	< LOQ	< LOQ	< LOQ
4-weeks	< LOQ	< LOQ	< LOQ	< LOQ

Blood samples were collected for 4-weeks post-injection from 8 dogs: 4 females (*f* = number of females detected with the metabolite) and 4 males (*m* = number of males detected with the metabolite). Data are presented as median (range as minimum-maximum).

LOQ, limit of quantification = 0.5 ng/mL.

### 3.3 Blood work

Hematology and serum chemistry data are presented in Table 4. White blood cells (WBCs) were significantly affected by L-sCBD injection (*p* = 0.008) at 3-days, with significant increase from baseline in neutrophil count (*p* = 0.007) and percentage (*p* = 0.039), significant increase in monocyte count (*p* = 0.016) and significant decrease in lymphocyte percentage (*p* = 0.023). Neutrophil count was above the reference interval in 2/8 dogs. Significant higher counts were also observed when L-sCBD was compared with placebo (*p* = 0.042 [WBCs], *p* = 0.023 [neutrophils] and *p* = 0.016 [monocytes]). At 1-week post injection, neutrophil count was still significantly higher than placebo (*p* = 0.016), however at 4-weeks no difference was observed. Red blood cell (RBC) variables showed a time effect on RBC count (*p* = 0.015) with declines in both groups (L-sCBD in −4.7%, placebo in −4.1%), hematocrit (*p* = 0.015; L-sCBD in −6.3%, placebo in −4.8%) and hemoglobin (*p* = 0.032; L-sCBD in −4.5%, placebo in −4.3%). Furthermore, L-sCBD showed significant lower values compared with placebo at 3-days post-injection (*p* = 0.042 [RBC], *p* = 0.008 [hematocrit] and *p* = 0.021 [hemoglobin]), and at 1-week (only hematocrit, *p* = 0.023). Although RBC variables were decreased at 3-days post-L-sCBD injection, only 2/8 dogs had RBC count below the reference interval, while hematocrit and hemoglobin were within the reference interval. Platelets showed a significant decrease from baseline at 3-days post-L-sCBD-injection (*p* = 0.014), which was also significantly lower than placebo (*p* = 0.023), but values remained within reference interval in all dogs.

**Table 4:** Complete blood count and serum chemistry results performed in eight dogs before and 4-weeks after subcutaneous injections of liposomal-synthetic-cannabidiol (L-sCBD; 7 mg/kg) or placebo (empty liposomes) in a randomized manner.

Analyte	Reference interval	Baseline	Treatment	3-days	1-week	4-weeks
Complete blood count						
WBC (10^3^/μL)	5.2–13.9	7.9 (6.8–11.7)	L-sCBD	12.3 (9.9–21.3)*^†^	9.2 (6.7–13.8)	8.0 (6.7–11.8)
			Placebo	9.8 (6.8–12.1)	7.6 (6.6–12.8)	7.8 (5.9–12.1)
Neutrophils (10^3^/μL)	3.9–8.0	5.3 (3.9–7.7)	L-sCBD	8.8 (6.2–17.8)*^†^	6.3 (4.1–10.4)^†^	4.9 (3.9–8.5)
			Placebo	7.1 (4.0–8.8)	4.7 (3.5–9.2)	4.8 (3.7–9.1)
Lymphocytes (10^3^/μL)	1.3–4.1	2.0 (1.6–3.7)	L-sCBD	2.1 (1.6–4.1)	1.8 (1.4–2.7)	1.8 (1.5–2.4)
			Placebo	1.9 (1.5–3.2)	1.9 (1.6–3.4)	1.9 (1.5–2.6)
Monocytes (10^3^/μL)	0.2–1.1	0.45 (0.23–0.49)	L-sCBD	0.69 (0.4–1.1)*^†^	0.54 (0.35–0.86)	0.36 (0.27–0.60)
			Placebo	0.44 (0.31–0.54)	0.45 (0.24–0.57)	0.37 (0.25–0.53)
Eosinophils (10^3^/μL)	0.0–0.6	0.47 (0.10–0.70)	L-sCBD	0.39 (0.16–0.54)	0.46 (0.30–0.62)	0.57 (0.26–0.91)
			Placebo	0.55 (0.18–0.85)	0.51 (0.34–0.99)	0.48 (0.27–0.88)
Basophils (10^3^/μL)	0.0–0.1	0.01 (0–0.02)	L-sCBD	0.01 (0.01–0.05)	0.01 (0–0.02)	0.01 (0–0.03)
			Placebo	0.01 (0–0.02)	0.01 (0.01)	0.01 (0–0.07)
Neutrophils (%)	42.5–77.3	62.7 (54.1–72.8)	L-sCBD	72.4 (52.0–84.4)*	67.8 (56.7–75.7)	64.3 (54.3–73.6)
			Placebo	66.4 (59.4–75.5)	64.5 (53.5–75.8)	62.3 (55.9–75.0)
Lymphocytes (%)	11.8–39.6	23.9 (18.9–32.8)	L-sCBD	17.4 (8.8–34.5)*	19.2 (13.2–30.9)	24.8 (15.9–30.3)
			Placebo	22.1 (15.3–26.6)	24.7 (16.8–31.6)	26.8 (13.1–32.2)
Monocytes (%)	3.3–10.3	5.3 (3.2–6.4)	L-sCBD	5.2 (3.3–8.9)	5.6 (4.6–10.1)	4.6 (3.5–6.3)
			Placebo	4.6 (3.3–5.7)	4.7 (3.6–6.7)	4.6 (3.4–6.4)
Eosinophils (%)	0.0–7.0	6.6 (1.0–9.6)	L-sCBD	2.5 (1.2–3.8)	4.7 (3.8–7.3)	7.3 (2.8–11.6)
			Placebo	5.8 (1.5–9.3)	6.1 (2.8–15.1)	5.7 (3.7–11.3)
Basophils (%)	0.0–1.3	0.1 (0–0.3)	L-sCBD	0.1 (0–0.1)	0.1 (0–0.2)	0.1 (0–0.5)
			Placebo	0.2 (0.1–0.6)	0.1 (0.1–0.2)	0.1 (0–0.7)
RBC (10^6^/μL)	5.7–8.8	7.5 (5.8–8.7)	L-sCBD	6.3 (5.3–7.1)*^†^	6.4 (5.1–7.4)*	7.1 (5.6–8.2)
			Placebo	6.6 (5.4–8.5)*	6.7 (5.3–7.7)*	6.7 (5.8–8.6)*
Hematocrit (%)	37.1–57.0	52.4 (43.4–58.7)	L-sCBD	41.0 (40.1–47.2)*^†^	44.0 (37.3–49.1)*^†^	48.4 (41.7–52.8)
			Placebo	48.2 (40.3–57.1)	47.4 (40.3–49.7)	48.1 (43.8–53.6)
Hemoglobin (g/dL)	12.9–18.4	17.4 (13.9–18.6)	L-sCBD	14.4 (12.3–15.8)*^†^	14.9 (11.7–16.3)*	16.4 (13.0–16.9)
			Placebo	15.7 (13.2–19.1)*	15.6 (12.8–16.4)*	15.4 (13.4–17.9)*
MCV (fL)	58.8–71.2	71.4 (59.6–76.5)	L-sCBD	69.4 (60.0–75.7)	70.2 (61.7–74.2)	70.9 (60.1–75.4)
			Placebo	71.3 (62.2–75.4)	70.4 (63.4–75.8)	70.4 (62.6–76.4)
MCH (pg)	20.5–24.2	23.2 (21.0–25.4)	L-sCBD	23.1 (21.2–25.4)	23.1 (21.0–25.1)	23.1 (20.7–25.4)
			Placebo	23.3 (21.2–25.4)	23.5 (20.8–25.9)	22.7 (20.9–25.0)
MCHC (g/dL)	31.0–36.2	32.7 (31.7–35.2)	L-sCBD	34.4 (30.6–35.8)	33.7 (31.3–35.5)	33.4 (31.7–35.0)
			Placebo	33.0 (31.9–35.0)	32.7 (31.2–34.8)	32.6 (30.5–34.7)
Reticulocytes (10^9^/L)	8–129	54 (11–107)	L-sCBD	23.9 (6.6–57.9)	19.4 (12.7–57.6)	25.2 (8.2–102.0)
			Placebo	50.0 (13.1–108.1)	28.8 (14.5–106.0)	39.6 (10.0–121.6)
Reticulocytes (%)	0.1–2.0	0.6 (0.1–1.5)	L-sCBD	0.4 (0.1–0.9)	0.3 (0.2–1.0)	0.4 (0.1–1.5)
			Placebo	0.8 (0.2–1.7)	0.4 (0.3–1.6)	0.6 (0.1–1.9)
Platelets (10^3^/μL)	143–400	212 (126–429)	L-sCBD	197 (121–335)*	283 (103–485)^†^	216 (98–301)
			Placebo	207 (157–407)	199 (165–381)	212 (164–397)
PCT (%)	0.1–0.4	0.2 (0.1–0.5)	L-sCBD	0.2 (0.1–0.4)	0.3 (0.1–0.6)*^†^	0.2 (0.1–0.4)
			Placebo	0.2 (0.2–0.5)	0.2 (0.2–0.5)	0.2 (0.2–0.5)
MPV (fL)	7.0–11.0	10.7 (10.2–11.3)	L-sCBD	11.9 (10.2–13.1)	12.3 (10.4–14.4)	10.9 (9.9–16.5)
			Placebo	11.6 (10.0–13.1)	11.0 (9.9–14.9)	11.2 (10.0–11.6)
PDW (%)	40.6–65.2	56.5 (47.7–68.0)	L-sCBD	58.1 (50.4–66.2)	56.6 (47.1–69.9)	55.7 (47.1–69.0)
			Placebo	53.7 (50.3–69.0)	56.9 (47.3–65.9)	51.5 (45.6–70.0)
Serum chemistry						
CK (IU/L)	51–399	100 (60–399)	L-sCBD	90 (50–203)	132 (56–319)	74 (52–152)
			Placebo	97 (63–281)	96 (60–312)	79 (62–320)
AST (IU/L)	19–42	28 (15–29)	L-sCBD	24 (13–31)	27 (21–33)	21 (13–27)
			Placebo	25 (15–31)	24 (20–31)	23 (14–32)
ALT (IU/L)	19–67	28 (11–97)	L-sCBD	21 (9–73)	26 (9–91)	25 (23–99)
			Placebo	30 (20–100)	31 (18–108)	28 (18–113)
ALP (IU/L)	21–170	49 (18–208)	L-sCBD	127 (77–317)*^†^	91 (45–303)*^†^	53 (28–208)
			Placebo	52 (20–203)	48 (23–218)	48 (22–254)
GGT (IU/L)	0–6	3 (3–7)	L-sCBD	3 (3)	3 (3–4)	3 (3–4)
			Placebo	3 (3–6)	3 (3–8)	3 (3–8)
Amylase (U/L)	103–1,510	458 (375–760)	L-sCBD	546 (506–1,029)	544 (413–942)	429 (380–734)
			Placebo	533 (452–730)	526 (322–847)	480 (391–956)
Triglyceride (mg/dL)	19–133	67 (39–141)	L-sCBD	52 (27–91)	73 (38–127)	71 (44–128)
			Placebo	66 (45–233)	69 (33–86)	86 (48–134)
Cholesterol (mg/dL)	135–361	245 (181–373)	L-sCBD	247 (175–379)	235 (154–396)	230 (174–353)
			Placebo	240 (186–379)	227 (194–381)	231 (183–395)
Total bilirubin (mg/dL)	0.0–0.2	0.15 (0.15)	L-sCBD	0.15 (0.15)	0.15 (0.15–0.2)	0.15 (0.15)
			Placebo	0.15 (0.15–0.18)	0.15 (0.15–0.16)	0.15 (0.15)
Glucose (mg/dL)	64–123	92 (62–106)	L-sCBD	90 (74–103)	81 (66–87)	93 (84–98)
			Placebo	85 (68–93)	86 (60–96)	86 (68–107)
Albumin (g/dL)	3.0–4.4	4.1 (2.7–4.6)	L-sCBD	3.4 (2.4–3.8)*^†^	3.7 (2.8–4.0)*	3.9 (2.6–4.7)
			Placebo	3.9 (2.7–4.6)	3.9 (3.1–4.6)	3.8 (2.6–4.5)
Total protein (g/dL)	5.4–7.6	6.6 (6.2–7.2)	L-sCBD	6.0 (5.7–6.8)*^†^	6.3 (5.6–6.6)*	6.4 (5.7–6.7)
			Placebo	6.4 (5.8–6.9)	6.4 (6.0–7.1)	6.5 (6.0–7.1)
Urea (mg/dL)	10.7–53.5	30.9 (28.0–53.1)	L-sCBD	28.6 (18.5–79.5)	31.7 (24.3–49.0)	32.9 (21.3–38.6)
			Placebo	31.0 (23.3–51.0)	36.8 (21.6–44.9)	33.1 (24.7–40.9)
Creatinine (mg/dL)	0.3–1.3	1.0 (0.8–1.2)	L-sCBD	0.9 (0.7–1.4)	1.1 (0.7–1.3)	1.0 (0.7–1.3)
			Placebo	1.0 (0.7–1.3)	1.0 (0.8–1.3)	1.0 (0.7–1.3)
Phosphate (mg/dL)	3.0–6.2	3.7 (3.1–4.7)	L-sCBD	3.9 (2.9–4.5)	4.0 (3.4–4.8)	3.4 (3.3–4.7)
			Placebo	3.7 (2.8–4.6)	3.7 (3.3–4.6)	3.5 (3.1–4.9)
Calcium (mg/dL)	9.7–11.5	10.5 (9.5–11.4)	L-sCBD	9.4 (9.2–10.8)*^†^	10.1 (9.2–11.5)	10.1 (9.3–10.9)
			Placebo	10.4 (9.3 – 11.7)	10.5 (9.3–11.9)	10.4 (9.6–11.7)
Sodium (mmol/L)	140–154	146 (140–151)	L-sCBD	142 (135–151)	145 (139–153)	143 (138–149)
			Placebo	144 (135–147)	145 (137–148)	145 (142–152)
Chloride (mmol/L)	104–118	111 (108–120)	L-sCBD	110 (104–117)	111 (104–120)	110 (108–120)
			Placebo	113 (106–115)	112 (109–116)	111 (109–117)
Potassium (mmol/L)	3.6–5.3	5.0 (4.7–5.9)	L-sCBD	4.5 (3.8–5.2)	4.9 (4.5–5.1)	4.6 (3.8–4.9)
			Placebo	4.8 (4.5–5.4)	4.8 (4.4–5.2)	4.6 (4.0–5.4)
CO_2_ (mmol/L)	16.0–26.0	19.5 (16.1–23.0)	L-sCBD	18.8 (14.1–20.1)	20.6 (16.8–23.1)	20.0 (17.1–24.6)
			Placebo	20.0 (15.0–21.2)	20.0 (13.3–22.6)	19.9 (16.6–22.7)

Data are presented as median (range as minimum-maximum). WBC = white blood cells; RBC = red blood cells; MCV = mean corpuscular volume; MCH = mean corpuscular hemoglobin; MCHC = mean corpuscular hemoglobin concentration; PCT = plateletcrit; MPV = mean platelet volume; PDW = platelets distribution width; CK = creatine kinase; AST = aspartate aminotransferase; ALT = alanine transaminase; ALP = alkaline phosphatase; GGT = gamma-glutamyl transferase.

*Significantly different from baseline value (*p* < 0.05).

^†^Significantly different from placebo (*p* < 0.05).

Serum chemistry showed a significant increase of alkaline phosphatase (ALP) at 3-days (*p* = 0.008) and at 1-week (*p* = 0.021) post-L-sCBD-injection, which was also significantly higher when compared to placebo (*p* = 0.008 and *p* = 0.014, respectively). Although increased, ALP was within reference interval in all dogs, except one dog where the baseline ALP was above the reference interval. Albumin, total protein and calcium were significantly decreased from baseline at 3-days post-L-sCBD-injection (*p* = 0.008). These analytes were also significantly lower than placebo at 3-days (*p* = 0.049, *p* = 0.008 and *p* = 0.014, respectively). Albumin values were kept within reference interval, except for one dog where the baseline albumin was below the reference interval. Total protein remained within reference interval in all dogs and calcium was decreased below the reference interval in 5/8 dogs. At 1-week post-L-sCBD-injection, albumin and total protein were still significantly decreased from baseline values (*p* = 0.034 and *p* = 0.008, respectively).

### 3.4 Body weight and vital signs

Body weight did not change significantly from baseline in any of the treatment periods. At 3-days post-L-sCBD injection, a significant RT increase from baseline was observed (*p* = 0.002), which was also significantly higher than placebo (*p* < 0.001; Table 5).

**Table 5:** Physiologic parameters and body weight from eight dogs at baseline and 4-weeks after subcutaneous injections of liposomal-synthetic-cannabidiol (L-sCBD; 7 mg/kg) or placebo (empty liposomes) in a randomized manner.

Variable	Baseline	Treatment	6-h	3-days	1-week	2-weeks	3-weeks	4-weeks
HR (bpm)	98 (80–120)	L-sCBD	86 (76–104)	102 (92–116)	84 (64–112)	94 (64–128)	94 (72–116)	86 (76–128)
		Placebo	84 (72–100)	96 (72–100)	86 (76–96)	92 (84–104)	96 (80–120)	90 (80–108)
*f*_R_ (rpm)^#^	32 (20–44) (*n* = 4)	L-sCBD	30 (20–40) (*n* = 4)	32 (24–40) (*n* = 4)	32 (20–40) (*n* = 6)	28 (24–36) (*n* = 5)	30 (28–32) (*n* = 4)	32 (28–36) (*n* = 4)
		Placebo	28 (20–44) (*n* = 4)	32 (20–36) (*n* = 5)	32 (24–36) (*n* = 4)	30 (28–32) (*n* = 4)	32 (24–36) (*n* = 6)	28 (20–36) (*n* = 4)
RT (°C)	38.7 (38.2–39.1)	L-sCBD	38.5 (38.0–38.8)	39.2 (38.4–39.8)*	38.4 (38.0–38.8)	38.4 (38.0–38.8)	38.5 (38.1–38.6)	38.6 (38.1–38.8)
		Placebo	38.4 (38.3–38.7)	38.5 (38.1–38.8)	38.4 (38.2–38.9)	38.7 (38.2–39.4)	38.4 (38.0–38.8)	38.6 (38.1–39.0)
MAP (mmHg)	109 (90–124)	L-sCBD	103 (95–112)	100 (96–106)	104 (90–114)	109 (94–127)	105 (94–109)	104 (96–113)
		Placebo	105 (96–118)	108 (102–114)	109 (104–113)	106 (94–122)	107 (100–117)	99 (90–121)
Body weight (kg)	33.5 (22.9–42.6)	L-sCBD	–	–	34.1 (22.5–42.5)	33.8 (22.5–42.7)	33.7 (22.7–42.7)	33.5 (22.7–42.5)
		Placebo	–	–	34.0 (22.3–42.6)	34.3 (22.3–42.0)	34.4 (22.1–42.7)	34.3 (22.1–42.7)

Data are presented as median (range; minimum-maximum).

HR, heart rate; f_R_, respiratory frequency; RT, rectal temperature; MAP, mean arterial pressure; bpm, beats per minute; rpm, respirations per minute.

^#^38–50% of the dogs were panting during physical exams, therefore n = number of dogs with countable respiratory frequency.

*Significantly different from baseline value and from placebo treatment (*p* < 0.05).

### 3.5 Behavior, activity monitoring collar, lameness and pain assessment

Subsequent to L-sCBD treatment, dogs showed significantly improved behavior compared to those in the placebo treatment (*p* = 0.0217). Specifically, 100% of dogs post-L-sCBD injection demonstrated improved function compared to only 25% post-placebo injection. The behavior/function reported by the owners included increased/resumed playing, climbing on the beds/sofa, improved climbing stairs and generally increased energy.

Efficacy data are presented in Table 6. None of the collected parameters from the activity monitoring collar changed significantly from baseline in any of the treatment periods and were not different between treatments. No significant difference in QoL scores was observed between treatments (*p* = 0.458). VLS was significantly lower (i.e., lameness decreased) post-L-sCBD injection *versus* the placebo treatment (*p* = 0.033). iVAS scores were significantly lower (i.e., less painful) post-L-sCBD injection *versus* the placebo treatment (*p* = 0.005).

**Table 6:** Weekly activity monitoring collar scores (PetPace), quality of life (QoL)^1^ scores by owners, visual lameness scale (VLS)^2^ by a small animal orthopedic surgeon, and interactive visual analog scale (iVAS)^3^ by two veterinary specialists: Surgery and anesthesia.

	Baseline	Treatment	Week 1	Week 2	Week 3	Week 4
PetPace						
Activity score	28 (19–51)	L-sCBD	28 (17–46)	32 (22–47)	30 (21–45)	28 (23–43)
		Placebo	32 (19–44)	31 (20–38)	30 (21–45)	32 (20–45)
Position score	658 (474–883)	L-sCBD	647 (436–713)	671 (514–838)	639 (524–837)	648 (505–808)
		Placebo	605 (512–862)	678 (489–813)	640 (456–809)	668 (409–762)
Sleep score (%)	82 (70–89)	L-sCBD	83 (79–90)	82 (77–88)	83 (76–88)	81 (77–89)
		Placebo	82 (76–87)	83 (76–87)	83 (75–88)	84 (71–88)
Calories expedite	1,229 (948–1,602)	L-sCBD	1,202 (958–1,614)	1,242 (901–1,585)	1,211 (971–1,595)	1,182 (990–1,584)
		Placebo	1,215 (962–1,567)	1,200 (945–1,627)	1,243 (946–1,592)	1,194 (965–1,625)
Pulse	66 (59–106)	L-sCBD	78 (60–101)	70 (55–97)	68 (54–97)	69 (54–87)
		Placebo	68 (61–94)	69 (59–94)	67 (57–91)	68 (60–91)
HRV	11.35 (10.92–11.65)	L-sCBD	11.26 (10.78–11.58)	11.35 (11.04–11.73)	11.36 (10.99–11.73)	11.37 (11.15–11.72)
		Placebo	11.36 (10.95–11.57)	11.29 (10.98–11.63)	11.34 (11.07–11.66)	11.35 (11.09–11.64)
Scorings						
QoL^1^	3.5 (3–4)	L-sCBD	4 (2–4)	3 (2–3)	2.5 (1–4)	3 (2–3)
		Placebo	3 (3–4)	3 (3–4)	3 (3–4)	3 (3–4)
VLS^2#^	2 (1–4)	L-sCBD	–	2 (1–2)*	–	2 (1–2)*
		Placebo	–	2 (1–3)	–	2 (1–3)
iVAS^3#^ (surgeon)	3 (2–6)	L-sCBD	–	1.5 (0–3)*	–	1.5 (0–3)*
		Placebo	–	3 (0–3)	–	3 (2–4)
iVAS^3^ (anesthesia specialist)	7 (6–9)	L-sCBD	6.5 (5–7)	5 (4–6)*	5 (4–6)*	6 (4–8)
		Placebo	6.5 (5–9)	6.5 (5–8)	6.5 (5–9)	7 (6–9)

Data were recorded from eight dogs with naturally-occurring osteoarthritis at baseline and 4-weeks after liposomal-synthetic-cannabidiol (L-sCBD; 7 mg/kg) and placebo (empty liposomes) subcutaneous injections in a randomized manner. Owners and veterinary evaluators were blinded to treatments. Data are presented as median (range; minimum-maximum).

HRV = heart rate variability.

^1^QoL scale 1–5; poor = 1, fair = 2, good = 3, very good = 4 and excellent = 5.

^2^VLS scale 0–5; 0 = sound; normal limb use, 1 = detectable lameness, 2 = mild weight bearing lameness, 3 = moderate weight bearing lameness, 4 = severe weight bearing lameness, and 5 = non weight bearing lameness.

^3^iVAS scale 0–10; 0 = no pain, 10 = worse pain.

^#^VLS and iVAS (surgeon) were scored at baseline and then every other week.

*Significantly improved from baseline and better than placebo (*p* < 0.05).

### 3.6 Adverse effects and follow-up

Adverse effects post-L-sCBD injection included fever (>39.0 °C) with significantly higher incidence than placebo (*p* = 0.006), decreased appetite in the first 2-days post-injection (*p* = 0.0263), and a local response (swelling) at the injection site (incidence 6/8 dogs; *p* = 0.007); (Table 7). The swelling resolved spontaneously (i.e., completely absorbed) within 5-8-days post-L-sCBD-injection. New morbidities or mortalities were not observed in any dog for at least 12-months after study completion. Individual follow-up information is presented in Table 7.

**Table 7:** Adverse local and systemic effects and follow-up of eight dogs with naturally-occurring osteoarthritis after liposomal-synthetic-cannabidiol (L-sCBD; 7 mg/kg) subcutaneous injection compared with placebo injection.

Dog	Fever^1^	Local response^2^	Follow-up
1	No	Yes	Per owner request, continued receiving L-sCBD injections every 4–10 weeks (total injections 16). Was diagnosed with anal sac carcinoma and lung metastasis 2 years & 3 months after study completion, and was euthanized due to deterioration in life quality 2.5 years after study completion, and 8-weeks after the last L-sCBD injection
2	Yes	None observed	Generally managed well with gabapentin addition. At the time of manuscript submission, 2 years & 10-months following injection
3	Yes	Yes	Generally managed well. 2.5 years after study completion, started bedinvetmab (Librela) injections. At the time of manuscript submission, 2 years & 9-months following injection
4	Yes	Yes	Several months after study completion, started bedinvetmab (Librela) injections, which helped for several months, then deteriorated and Librella discontinued. The dog was euthanized 1 year & 8-months after study completion
5	Yes	Yes	After study completion oral CBD oil was initiated, but without beneficial effect. Limb amputation performed 4 months after study completion (hind-limb mass increased in size). Lung metastases were detected 6-months later. The dog was euthanized 12-months after study completion
6	Yes	None observed	Generally managed well, primarily due to significant weight loss (42.6 to 34 kg; moved to Hill’s Prescription Diet, Metabolic). At the time of manuscript submission, 12-months following injection
7	Yes	Yes	Generally managed well with oral CBD oil addition. At the time of manuscript submission, 12-months following injection
8	Yes	Yes	Generally managed well with oral CBD oil addition. Lateralization surgery performed after study completion due to laryngeal paralysis (diagnosed before study enrolment). At the time of manuscript submission, 12-months following injection

^1^Fever resolved spontaneously within 2-days.

^2^Local response (swelling at injection site) resolved spontaneously within 5–8 days.

**Figure 3:**
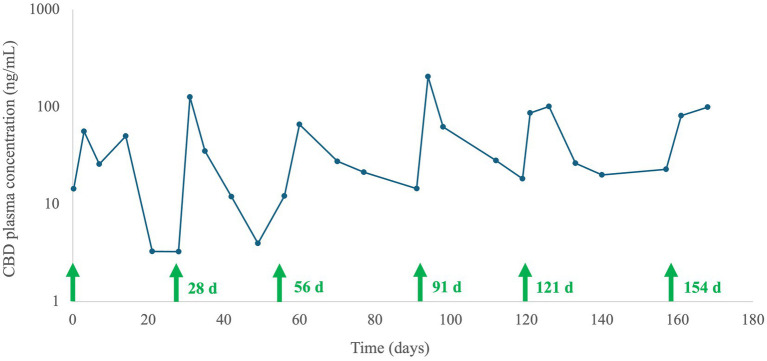
Cannabidiol (CBD) plasma concentrations (ng/mL) from one dog after multiple liposomal-synthetic-CBD (L-sCBD; 7 mg/kg) subcutaneous injections (a total of 6 injections). The first injection was administered on day 0, and the exact administration day (d) of the following injections is marked with green arrows.

One of the dogs (#1) showed marked improvement in mobility subsequent to L-sCBD treatment, and the owners requested to continue with the L-sCBD injections after study completion. This dog was monitored closely following 5 additional injections administered every 4–5 weeks over a period of 20-weeks, and CBD plasma concentrations for the duration of the repeated injections are presented in Figure 3. Interestingly, the trough concentrations of CBD increased over time, and the concentration-time curve seemed to become flatter, which may indicate approaching steady-state. Local swelling at the injection site was the only adverse effect observed during that period. Later the dog continued to receive the injections every 6–10 weeks (per owner request according to deterioration in dog’s mobility) without sampling for PK. A total of 16 injections were performed over 2.5 years, during which CBC and serum chemistry taken prior to each injection were unremarkable. However, 5–6 days following two out of the 16 injections, exudate was seen draining from the local swelling. A sample of the exudate was submitted for culture and sensitivity which was negative for bacterial growth. This adverse reaction was managed conservatively for several days and resolved completely within a week of presentation.

## 4 Discussion

### 4.1 Pharmacokinetics

Results from the present study showed that a single subcutaneous L-sCBD injection provided long-term (i.e., a minimum of 4-weeks) CBD plasma concentrations. Compared with six dogs administered a lower single-dose of 5 mg/kg L-sCBD, the median C_max_ (45.2 [17.8–72.5] ng/mL) and AUC (490 [189–803] ng·d/mL) were higher in the present study, while the median T_max_ (4 [2–14]) days was similar (16). When the median AUC was normalized to dose (AUC per mg/kg dose administered), both studies showed the same AUC (98 *versus* 99 ng·d/mL/mg/kg), suggesting proportional increase in exposure with dose increase. In the present study some variability in C_max_ was observed, which can be attributed to differences in metabolism related to age and sex or to absorption from injection site and differences in subcutaneous adipose tissue (25, 26). When comparing the results of the present study and the study where 5 mg/kg L-sCBD was injected, it seems that the effect of increasing L-sCBD dose on C_max_ is positive, as was reported in horses after oral CBD administration (27). However, ultimately the maximal concentration of CBD achieved from liposomal formulation depot is limited by the slow rate of absorption from the injection site.

Samara et al. reported in 1988 an intravenous CBD administration (2.25–4.5 mg/kg) in dogs, but the CBD was dissolved only in 70% alcohol (28). A recent study in beagle dogs reported the pharmacokinetics of intravenously administered 2.2 mg/kg CBD dissolved in medium-chain triglycerides (MCT oil) and further diluted in 70% ethanol (29). The dose-normalized AUC reported in that study was 98.4 ng·d/mL/mg/kg (29), which is the same as the dose-normalized AUC observed in the present study (99 ng·d/mL/mg/kg). This suggests that bioavailability of the L-sCBD was 100% and superior to the bioavailability reported in the same study using a single-dose oral CBD-MCT oil, where the measured oral bioavailability was reported to be 31% (29). Injectable liposomal formulations have the benefit of improving bioavailability by passing the liver and reaching directly into the blood. Additionally, oral bioavailability was reported to be significantly affected by fasted or fed states. In people, presence of high-fat content food in the gastrointestinal tract increase CBD absorption (30), which is thought to be attributed to favorable lymphatic transport (31).

In the present study half-life was several days long, but was shorter than the median half-life reported in dogs injected with L-sCBD at 5 mg/kg (12.4 [7.7–42.6] days) (16). The difference may be attributed to the longer time used for the calculation of half-life, which was 6-weeks in the previous study compared with 4-weeks in the current study. When half-life was calculated for the 3 dogs with longer available CBD data, then similar half-lives were observed. Another reason for the difference may be related to the different dose administered; in a study in three minipigs injected with 5, 7.5 or 10 mg/kg of L-sCBD (a single dose per minipig), the half-life decreased with increasing the dose; 8, 6.1 and 4 days, respectively (32). When a drug is absorbed slowly from a depot formulation, the terminal half-life is dependent on the rate of absorption, and not on drug’s elimination rate. This is described as “flip-flop” pharmacokinetics (26), and the extended duration of CBD plasma concentrations following L-sCBD injection fits such a kinetic profile. When comparing the half-life of immediate-release formulations, such as oral formulations, to the half-life of liposomal slow-release formulations, one should acknowledge that two different processes are compared (elimination *versus* absorption rate). Half-lives after a single dose of 2 mg/kg oral CBD in dogs was reported to be several hours in most studies; 4.2 h (6), 2.5 h (33), 1.0 h (34), and 3.8 h (35). In a study investigating 2.2 mg/kg CBD dissolved in MCT via intravenous and oral routes, half-life was reported to be 4.9 and 7 h, respectively (29). Higher doses of oral CBD at 5 and 8.3 mg/kg resulted in higher half-lives of 8.5–10.2 h (36) and 15.7 h (37), respectively.

CBD undergoes extensive metabolism in dogs with small quantity of unchanged CBD excreted in the urine (29). Two studies in dogs reported detection of CBD metabolites following oral CBD at 1–12 mg/kg, with 7-COOH-CBD being the primary metabolite, although peak concentration was lower than the parent drug, and 7-OH-CBD was detected in low concentrations (35, 38). In a recent study in beagle dogs administered 2.2 mg/kg of MCT-CBD intravenously and orally, CBD concentrations were the highest, followed by 7-COOH-CBD concentrations, while 7-OH-CBD had the lowest concentrations; C_max_ of 7-COOH-CBD and 7-OH-CBD was 47.7–49.7 and 7.0–7.4 ng/mL, respectively, with no differences between the intravenous and oral routes (29). Similarly, the results of the present study showed that the parent drug produced the highest plasma concentrations, followed by 7-COOH-CBD, while 7-OH-CBD produced the lowest concentrations. In another study in dogs administered 2, 5 or 10 mg/kg oral CBD (cannabis herbal extract containing THC: CBD at 1:20 ratio), 6-OH-CBD was reported to be the primary CBD metabolite with C_max_ increasing with dose (33). In the present study, 6α- and 6β-OH-CBD metabolites were also detected, and their concentrations were lower than the parent drug. Additionally, in the present study for each individual dog, CBD metabolite formation seems to correlate directly with CBD plasma concentrations (i.e., metabolites were detected when CBD concentrations were higher). Interestingly, all metabolites were detected and persisted longer in females when compared to males where not all metabolites were detected. This may indicate sex differences in CBD metabolism in dogs, as was previously reported in people (39). This finding is thought to be related to differences in adipose tissue disposition between men and women (39).

In minipigs, the major metabolite post-L-sCBD-injection was reported to be 7-COOH-CBD. Nevertheless, in contrast to dogs, its concentrations greatly exceeded those of the parent drug, and the AUC ratio of 7-COOH-CBD to CBD was 19–100 (32). The high AUC ratio of 7-COOH-CBD to CBD in minipigs is similar to the high ratio (35-fold) that is seen in people (40).

### 4.2 Blood work

The increased WBC count 3-days post-L-sCBD-injection was clinically the most important change observed, with two of the dogs exceeding the reference interval. This increase resulted from an increase in neutrophil count, and seemed to correlate with the local response observed at the injection site. The increased neutrophil count did not occur following the placebo injection, and we speculate that it presents an immune system response to the liposomal-CBD combination or to the CBD molecule itself. Hematocrit, hemoglobin, RBCs and platelets were decreased 3-days post-L-sCBD injection, but the decrease was mild and within reference interval in most dogs. In a former study in six dogs injected with L-sCBD at 5 mg/kg, WBCs increased in 3/6 dogs at 1-week post-injection, but this was the first sampling timepoint, therefore the increase may have been missed in some of the dogs (16). In the same study, RBC variables were decreased significantly at 1-week post-injection, but platelet count was increased significantly (16).

Several studies report significantly increased alkaline phosphatase (ALP) from baseline following administration of oral CBD in dogs (29, 34, 38, 41, 42). One of these studies reported that this elevation correlated with the elevation of bone-specific ALP, suggesting that ALP increase is potentially related to osteoblastic activity (42). Another study in dogs reported that the increase in ALP was dose-dependent, and the highest ALP concentrations were recorded following a dose of 12 mg/kg compared with lower doses (38). In the present study a mild, albeit significant ALP increase at 3-days post-L-sCBD-injection was observed, which decreased quickly and returned to baseline values by 4-weeks post-injection. Other liver enzymes, such as aspartate aminotransferase (AST), alanine transaminase (ALT) and gamma-glutamyl transferase (GGT) were not elevated in the present study. In people consuming CBD, these enzymes were reported to increase substantially from baseline, and were over 3-fold of the upper reference interval (43). In the present study albumin decreased significantly, although within the reference interval. This observation was also observed in dogs injected with 5 mg/kg L-sCBD (16). Another study in dogs reported a gradual albumin decrease during 6-months oral CBD administration (42). Further investigations of L-sCBD effects on liver function and enzymes in dogs are important as part of the safety assessment of this formulation.

### 4.3 Efficacy

The endocannabinoid system has an important role in neuromodulation and synaptic plasticity, and among other effects it can lead to analgesia. CBD is considered to have a complex analgesic mechanism of action, because it does not pose a direct agonistic effect on the endocannabinoid receptors, CB1 and CB2 (44, 45). Nevertheless, various mechanisms have been reported to be involved in its analgesic effects. Interactions with serotonin receptors (primarily *via* 5-HT1_A_ receptor), dopamine receptors and activation of transient receptor potential (TRP) ion channels (e.g., TRPV1, TRPV4, TRPM8 and TRPA1) (25, 45–49). Interactions with many membrane G protein-coupled receptors (GPCR), including antagonist effect on GPR55, and inverse agonist effect for GPR3, GPR6, GPR12 and GPR18, and interaction with *μ*- and *δ*-opioid receptors (25, 47–49). With regard to inflammation, an important target of CBD is the nuclear receptor peroxisome proliferator-activated receptor gamma (PPARγ) (25, 47, 49). Inhibition of COX2 (25, 48) and voltage-gated calcium channels (Ca_v_3.1 and Ca_v_3.2) on nociceptive neurons (45, 47, 50) have also been reported. In recent years CBD was also reported to block voltage-gated sodium-channels, including channels that are involved in nociception (Na_v_1.3 and Na_v_1.7), thereby, suggesting an additional mechanism of action of CBD as an analgesic (51–53).

Analgesic properties of CBD for conditions associated with chronic pain have been reported previously in experimental animal models (54), and clinically in people (55, 56), horses (57) and dogs (6–10). Our previous study in dogs with naturally-occurring osteoarthritis injected with L-sCBD (5 mg/kg) showed significant improvement in pain assessments post-injection. However, this was an open-label study, which can result in evaluator bias (16). The present study was designed with a placebo-controlled limb in order to reduce bias. Significant positive effects on pain reflected in reduced lameness and pain scores and improved behavior were found when compared with the placebo injection. A placebo effect of 25% (2/8 dogs) was observed in the present study by both owners and the primary veterinary caregiver. This effect is lower (i.e., better ability to differentiate improvement) than a placebo effect of 40–45% reported for osteoarthritic dogs assessed for lameness by their owners and caregivers (58). An interesting description of dogs’ behavior improvement by owners was that the dog resumed a playful behavior. Playfulness could be a good sign or monitoring end-point of pain alleviation in osteoarthritic dogs, although this assumption should be further investigated and validated.

Several additional differences exist, when this study is compared to other CBD placebo-controlled studies in dogs with osteoarthritis-associated pain. The CBD in L-sCBD is synthetic, compared to “CBD-rich hemp” administered in other studies. Hemp oil contains other cannabinoids, such as flavonoids and terpenoids, which may provide some anti-inflammatory properties by themselves (59), and can potentially exhibit cannabis synergy, known as the “entourage effect” (3, 60, 61). In addition, a crossover design was used in the present study in order to decrease the variability among subjects with each dog serving as its own control. This design has clear advantage over the use of two or more parallel treatment groups. Furthermore, the PetPace activity monitoring collar was used in the present study to provide objective data as previously reported in a study in osteoarthritic dogs (62). However, PetPace collars have several limitations which may introduce bias into the results. These collars cannot account for an owner’s decision-making (i.e., taking the dog on short *versus* long walks), and cannot differentiate between a car drive and other dog movements. Unfortunately, the use of recommended, accurate and validated tools for gait evaluation, such as force-plate or pressure sensitive walkway analysis (63), were not available in the present study.

A systematic review and meta-analysis considering efficacy of supplemental treatments (enriched diets and nutraceuticals) for osteoarthritic dogs and cats has recently been published (64). The authors assessed 9 different categories of natural supplemental treatments, including CBD nutraceuticals (CBD-rich hemp), and concluded that clinical analgesic efficacy was primarily evident for omega-3-based nutraceuticals and omega-3-enriched diets, followed by CBD-based nutraceuticals (64). Another systematic review and meta-analysis specifically considering administration of CBD in dogs with osteoarthritis, concluded that CBD administration may reduce pain severity scores and was generally well-tolerated, however, very low certainty of evidence and high risk of bias were reported (65). Therefore, high-quality placebo-controlled clinical trials should be further conducted in order to provide good evidence for recommending the use of CBD in canine osteoarthritis.

### 4.4 Safety

In the present study, a single injection of L-sCBD was tolerated by all dogs, and multiple injections administered in one of the dogs were generally well tolerated. The two primary adverse reactions observed included a 2-days short-term fever accompanied by inappetence and a local response at the injection site. A similar local response was also reported in 5/6 dogs injected with L-sCBD at 5 mg/kg (16). The local swelling was self-limiting in nature and resolved without any treatment. Liposomes are characterized by a great biocompatibility, due to their lipid components that are a normal part of the mammalian cells, and are biodegradable by lipases in the body. Liposomes were shown to have good safety profile and are not likely to induce foreign-material body reactions (19). The fact that in the present study no local response occurred after any of the empty liposomes-placebo injections, strengthen the biodegradable property of liposomal delivery systems. Therefore, the local response and the fever observed in the dogs of the present study are likely due to immune system responses to the liposomal-CBD combination or to the CBD molecule itself.

Common systemic adverse effects of oral CBD administration in dogs were primarily reported to be gastrointestinal related, such as loose feces or diarrhea, nausea, vomiting and hypersalivation (8–10, 38, 41, 66, 67), and less commonly sedation (66). None of these adverse effects were observed in the present study, nor in a former study of L-sCBD in dogs (16). Systemic adverse effects are usually related to high C_max_, therefore, the property of liposomal-based formulations to lower C_max_ can result in decreased toxicity of the encapsulated drugs (19, 68). The design of CBD encapsulated into liposomes took advantage of the high lipophilic nature of CBD (12), resulting in absorption rate that is slower than the elimination rate (i.e., “flip-flop” kinetics), thereby limiting the potential of high C_max_ and systemic adverse events (26).

### 4.5 CBD source

CBD formulations extracted from *Cannabis sativa* plants can vary substantially due to various factors affecting the composition of the formulation. These include different phytocannabinoid profiles among plant genotypes, and secondary metabolite accumulation due to growth conditions, storage or delivery route (69). It should be noted that in most cases the herbal products are characterized for CBD and THC only and not for the other components, which is very problematic from pharmaceutical perspectives. Additionally, different extraction methods were reported to affect drug composition and activity (70). One of the advantages of L-sCBD formulation is that it contains synthetic CBD, which is THC-free and characterized in pharmaceutically accepted standards. A controlled synthesis of manufactured CBD offers a more reliable and consistent pharmacologic profile, which can lead to predictable outcomes. Products made without THC are particularly safer for dogs, because THC can cause moderate to severe neurological adverse events in this species, as was reported following CBD/THC (20,1, at 10/0.5 mg/kg) (33) and following escalating doses of THC and CBD/THC (1.5/1) oils, but not following escalating doses of CBD oil (66).

### 4.6 Limitations

Limitations to this study include the generally small number of dogs completing the study, precluding stratified or covariate-adjusted analyses of sex and age. A major limitation was lack of validated objective assessment tool of lameness, such as force-plate or subjective validated tools, such as specific osteoarthritis owner-questionnaire. Although the study was designed to be blinded, in some of the dogs the local response was obvious and may have introduced bias to the owner and anesthesiology specialist evaluations (the surgery specialist met the dogs every-other-week, when the swelling was already absorbed). In dogs administered the L-sCBD injection first, a positive effect was kept to some degree into the placebo period, likely due to retained CBD plasma levels. In retrospect, a washout period of 4-6-weeks after the 4-week monitoring period between the 1st injection and administration of the 2nd injection was necessary in order to reduce the bias of a positive effect exceeding 4-weeks. Bioavailability was calculated based on results from another study, as intravenous formulation of CBD was not available for comparison in the present study. Another possible bias is a potential drug-interaction of the CBD with the different routine analgesic that each dog was receiving, as previously reported (71). An important example, is CBD potentiation of the analgesia produced by gabapentin (25, 49), a drug that 2/8 dogs were receiving.

## 5 Conclusion

Subcutaneous injection of L-sCBD in dogs with osteoarthritis-associated pain resulted in detectable CBD plasma concentrations for 4–8 weeks, and provided a significant positive effect on pain, lameness, and behavior when compared with placebo injection. Bioavailability of CBD seemed to be complete *via* this route, based on intravenous CBD administration in dogs (29).

Adverse effects of L-sCBD were tolerated by all dogs, however, additional cohort investigations with larger numbers of dogs and a longer duration of treatments would provide more information as to the long-term efficacy and safety of this formulation. The use of L-sCBD as an additional treatment as part of multimodal analgesia to increase wellbeing and quality of life in dogs suffering from osteoarthritis is of interest.

## Data Availability

The raw data supporting the conclusions of this article will be made available by the authors, without undue reservation.

## Appendix

Plasma concentration analysis of cannabidiol (CBD) and its metabolites by UHPLC–MS/MS.

**Table:**

Name	Precursor (*m*/*z*)	Product type	Product mass (*m*/*z*)	DP (V)	CE (eV)	CXP (V)	Rt (minute)
MRM transitions – CBD and CBG in positive ion mode							
CBD	315.1	Quantifier	123.1	40	43	18	10.65
		Qualifier	193.1	40	34	20	
CBG	317.1	Quantifier	123.1	80	43	14	10.55
		Qualifier	193.1	80	23	24	
MRM transitions – CBD metabolites and CBG in negative ion mode							
7-OH-CBD	329.1	Quantifier	268.2	−40	−36	−11	7.65
		Qualifier	299.0	−40	−22	−29	
6α-OH-CBD	329.1	Quantifier	158.2	−130	−40	−21	7.48
		Qualifier	173.2	−130	−34	−21	
6β-OH-CBD	329.1	Quantifier	173.2	−130	−34	−21	7.78
		Qualifier	158.2	−130	−40	−21	
7-COOH-CBD	343.0	Quantifier	231.0	−55	−34	−23	7.50
		Qualifier	179.1	−55	−32	−17	
CBG	315.1	Quantifier	191.2	−80	−32	−7	10.55

Cannabigerol (CBG) was used as internal standard (IS). Multiple reaction monitoring (MRM) transitions and variables for CBD, CBD metabolites and CBG (IS) in positive or negative ion modes. m/z = mass to charge ratio; DP = declustering potential; CE = collision energy; CXP = collision cell exit potential; V = volts; eV = electronvolts; Rt = retention time.

7-hydroxy-CBD, 7-OH-CBD; 6α/6β-hydroxy-CBD, 6α/6β-OH-CBD; 7-carboxy-CBD, 7-COOH-CBD.
